# Factors associated with consistent condom use: a cross-sectional survey of two Nigerian universities

**DOI:** 10.1186/s12889-019-7543-1

**Published:** 2019-09-02

**Authors:** Anthony Idowu Ajayi, Kafayat Olanike Ismail, Wilson Akpan

**Affiliations:** 1 Population Dynamics and Reproductive Health Unit, African Population and Health Research Center, APHRC Campus, Off Kirawa Road, Nairobi, Kenya; 2 Department of Sociology, Faculty of the Social Sciences, University of Ilorin, Ilorin, Nigeria; 3 Research and Innovation, Walter Sisulu University, Nelson Mandela Drive, Mthatha, South Africa

**Keywords:** Condoms, Self-efficacy, Consistent condom use, HIV, Drug use, Alcohol use, Nigeria, University students

## Abstract

**Background:**

Consistent condom use is central to the prevention of transmission of human immunodeficiency virus (HIV) and other sexually transmitted diseases (STDs), especially among young adults. This study drew from a cross-sectional survey of two Nigerian universities to determine the level of consistent condom use, explored the determinants of condom use consistency and reasons for inconsistent condom use.

**Methods:**

We adopted a descriptive cross-sectional design, which involves the recruitment of 800 male and female students using stratified random sampling. Adjusted and unadjusted logistic regression models were used to examine the factors associated with consistent condom use among a final sample of 498 students who engaged in sex in the last year preceding the study.

**Results:**

Only 38.6% of sexually active participants (*n* = 498) used condoms consistently in the previous year. High condom self-efficacy score (AOR: 2.40; 95% CI: 1.58–3.64), discussion of HIV/STIs with sexual partner (AOR: 1.91; 95%CI: 1.29–2.83), knowing partner’s HIV status (AOR: 1.48; 95% CI: 1.02–2.16), being students of university located in a high HIV prevalence area (AOR: 2.86; 95% CI: 1.92–4.28) and engaging in sex with only steady partner (AOR: 1.74; 95% CI: 1.17–2.60) were associated with a higher odds of consistent condom use. Trust, unavailability of condoms, dislike of condoms and a perception that condoms reduced sexual pleasure were the main reasons for inconsistent use of condoms.

**Conclusion:**

The study found a low level of consistent condom use among study participants. Counselling young adults in Nigeria on condom self-efficacy, providing condoms on campuses and encouraging the discussion of sexually transmitted infections with sexual partners are central to improving the level of consistent condom use among Nigerian university students.

**Electronic supplementary material:**

The online version of this article (10.1186/s12889-019-7543-1) contains supplementary material, which is available to authorized users.

## Background

Consistent condom use is central to the prevention of transmission of human immunodeficiency virus (HIV) and other sexually transmitted diseases (STDs), especially among young adults [1, 2]. In sub-Saharan Africa, HIV transmission still constitutes a public health concern, with many adolescents and young adults engaging in unprotected sex and transactional sex and reporting inconsistent condom use [3–10]. Globally, consistent condom use rates range from 4 to 52.4% among young, sexually active individuals [9–14]. Due to inconsistent condom use, young adults are at a higher risk of acquiring HIV/STIs. Not surprisingly, young adults account for over 50% of new HIV transmission [15–17]. Studies have also shown that young women are particularly susceptible to HIV acquisition due to social, cultural, economic and structural risk factors that predispose them to engage in risky sexual behaviour [18–20]. This calls for creative strategies to increase the rate of consistent condom use among young people. We defined consistent condom use in this study as the use of condoms at every sexual encounter in the last year.

Even though the prevalence of HIV is relatively low (3.2%) in Nigeria [21], this low prevalence translates into a large number of people living with HIV given the country’s large population. The country has the second-largest number of people living with HIV, after South Africa [21]. Although there are no nationally representative data on the prevalence of sexually transmitted diseases, the available evidence (mostly hospital-based studies) shows that the prevalence of STDs is high (14–18.8%) in Nigeria, especially among young people [22–27]. A study conducted in southeast Nigeria shows that the prevalence of STIs among females was as high as 17%, with age group 17–19 years more likely to have any type of STI infections (44%) [28]. What is more worrisome is that many young people are unlikely to seek care or seek care at informal sources, with males commonly seeking care from traditional healers [22–27].

Reasons for inconsistent condom use are well documented. These include low perceived sexual satisfaction derived from the use of condoms, low perceived personal risk of STDs, condom fatigue, having a steady sexual partner, and low condom self-efficacy [11–13, 29, 30]. Studies have identified self-efficacy as the most important predictor of condom use [13, 31–34]. Bandura [35] coined the term ‘self-efficacy’ and defines it as a “judgment of one’s capability to accomplish a certain level of performance”. The term is now in popular use in health behaviour and health promotion research, with condom self-efficacy referring to the belief that an individual would feel constantly confident in his/her ability to use a condom with a sexual partner without feeling embarrassed or rejected. Previous studies have reported that high condom self-efficacy has a strong influence on consistent condom use [10, 12]. However, this link is unclear among women [12, 36]. On the contrary, females are at a higher risk of inconsistent condom use, and this includes women who might have rated themselves highly in condom use self-efficacy [33].

The reasons young adults engage in unsafe sex practices despite knowledge of their consequences continue to generate scholarly interest. A few studies have examined the factors associated with consistent condom use in the Nigerian context (41–42). Onayade et al. (2008) conclude that younger age, having more than one sexual partner, and ability to refuse sex with a partner who does not want to use condoms was associated with a higher likelihood of consistent condom use among males. While the frequency of sexual intercourse and having more than one sexual partner were associated with a higher likelihood of consistent condom use among females. In contrast, Van Rossem et al. (2001) assert that awareness of the efficacy of condoms in preventing HIV and unwanted pregnancy and concerns about HIV and unwanted pregnancy were associated with a higher probability of consistent use of condoms (42). Overall, factors associated with consistent condom use in Nigeria are poorly understood, as there is a dearth of studies examining the influence of socio-demographic, behavioural and psychosocial factors.

## Hypotheses

Studies examining factors associated with consistent condom use among sexually active young people in Nigeria are scarce. To fully understand factors influencing consistent condom use among adolescents and young adults in the Nigerian context and also based on our review of the existing literature on the topic, we proposed five hypotheses. First, studies have established that condom self-efficacy is associated with consistent condom use [10, 12]. However, the links between condom self-efficacy and consistent condom use have never been investigated in Nigeria. We expect that individuals who expressed higher condom use self-efficacy are more likely to use condoms consistently. We, therefore, expressed our hypothesis as follows:

### Hypothesis 1

High levels of condom self-efficacy are associated with higher odds of consistent condom use among adolescents and young adults in Nigeria.

Also, knowing one’s partner status has been shown to promote HIV testing and safe sex practices [37]. While no studies have examined this effect on consistent condom use, we expect that individuals who know their partner’s status to be more likely to use condoms consistently compared to those who do not. This leads us to propose our hypothesis two:

### Hypothesis 2

Knowing one’s partner status is associated with higher odds of consistent condom use.

More so, there is evidence that couple communication about HIV/STIs promotes safe sex practices and HIV testing [37]. While there are studies that have examined the rate and correlates of consistent condom use in Nigeria [8, 30, 38–40], no studies have examined whether partner communication about HIV/STIs influences consistent condom use. Given the known effect of couple communication on safe sex practices and HIV testing, we expect that partner communication about HIV/STIs will likely increase the odds for consistent condom use. We expressed our hypothesis as follows:

### Hypothesis 3

Partner communication about HIV/STIs is associated with a higher likelihood of consistent condom use.

Also, given the concentration of programmes to reduce HIV transmission in settings with a disproportionately higher prevalence of HIV in Nigeria, we expect that individuals residing in high HIV prevalence areas to use condoms consistently compared to those residing in low prevalence areas, with little or no risk reduction interventions. Considering that this study was conducted in two settings— one, where the prevalence of HIV is high and the other where the prevalence of HIV is low— we assessed whether students from high HIV area are significantly more likely to use condoms consistently compared to those from areas with a low HIV prevalence. Our hypothesis is expressed as follows:

### Hypothesis 4

Students from areas with high HIV prevalence are significantly more likely to use condoms consistently compared to those from areas with a low HIV prevalence.

The finding on the relationship between partner type and consistent condom use is unclear. There is evidence that having sex with only a steady partner is associated with consistent condom use [41]. However, a study also shows that having sex with just a regular partner is associated with inconsistent condom use [39], while another study reported no significant difference [40]. We thus aim to examine the relationship number and type of sexual partners and consistent condom use among mostly unmarried young adults in Nigeria. We expressed our hypothesis as follows:

### Hypothesis 5

Having sex with only one partner is associated with a higher likelihood of consistent condom.

## Methods

### Participants and sampling

The data analysed in this study were part of a more extensive study that sought to examine reproductive health and wellbeing of university students in Nigeria [37]. This cross-sectional study was conducted using a researcher-administered questionnaire at the University of Ilorin and Nasarawa State University, both in North Central Nigeria and with a combined student population of approximately 45,000. The choice of a federal (national) and a state (provincial) university was purposive. Nasarawa State University was purposively selected due to its location in a state with one of the highest rates of HIV prevalence in Nigeria. The Northcentral region of Nigeria, where this study was conducted, has the highest HIV prevalence rate [21]. It is worth noting that Nasarawa State shares boundaries with the Federal Capital Territory, Benue State, Taraba State and Plateau State and that this region has the highest HIV prevalence rate in the country. The prevalence of HIV is low in Ilorin and environs [21]. The University of Ilorin, also located in the North central region of Nigeria, was selected because it is a federally-owned university and one of the highly sought-after universities in Nigeria. As a result, students are admitted to the university from all parts of Nigeria. In terms of tuition fee, both universities are similar; however, the inclusion of both universities further increases the diversity of students included in this study. The detailed methodology has been published elsewhere [42, 43].

A pilot survey (using a semi-structured questionnaire) was conducted among 20 participants. These participants were not included in the main study; instead, their feedback was used to improve the questionnaire. The pilot study took place at the University of Ibadan and was conducted mainly to test the validity and reliability of the instrument.

Sample size estimation was done using a web-based sample size calculator [44]. The appropriate sample size was estimated based on a prevalence of HIV testing uptake of 50% among the young, educated adults reported in a previous study [45]. For the sample size to detect a 5% error and representative of each school, a sample size of 384 is required. However, the appropriate sample size representative of each university at a confidence interval of ±5 and a 95% confidence level is 400 participants, after adjusting for possible missing responses. Overall, a pre-tested questionnaire was administered to 800 male and female students selected using a stratified sampling technique. Only 784 respondents returned a completed questionnaire. The survey took place in the two Nigerian universities between March and May 2018. The target population was stratified into both males and females, with proportional samples selected from both strata. For inclusiveness, respondents were also stratified by level of study and faculty of study. A random sample of eligible participants corresponding to the sizes of the strata was recruited.

In a culturally sensitive environment like Nigeria, researching sexual behaviours requires a high degree of privacy, confidentiality and trust. As such, research assistants were taken through a three-day training session, focusing on interview techniques, fieldwork ethics and the importance of earning study participants’ trust. The research assistants administered the questionnaire to consenting students in private spaces on campus and students’ rooms within the campus (for those who reside on campus residence) and outside of campus (for those who reside in private accommodations not far away from campus). Our preference for the interviewer-administered questionnaire is predicated on the advantage of this method concerning reducing missing responses. It is, however, worth noting that some participant opted to complete their questionnaire alone to which the research assistants permitted. Only undergraduate students of the selected university were included. Visiting students from another university and postgraduate students were excluded from the study.

The analysis in this study was limited to those who had reported being sexually active in the last year preceding the survey (*n* = 498). The study protocol was reviewed and approved by the Ethical Committees of the University of Fort Hare and Ondo State Ministry of Health. Each participant signed an informed consent form. Overall, the inclusion of participants was voluntary and willful. Respondents were guaranteed utmost confidentiality, privacy and anonymity. The study was conducted in line with the relevant national and institutional committees’ rules on human experimentation and with those of the Helsinki Declaration of 1975. Parent permission and assent were obtained for a few students (57) who were 17 years at the time of the study.

### Measures

#### Consistent condom use

Consistent condom use was measured using a dichotomised response format and open-ended questions, which allowed for the effective capturing of unstructured responses. To elicit responses regarding the consistency of condom use in recent penetrative sex, we asked sexually active participants four questions, which were: *“Did you or your boy/girlfriend use protection regularly in every sexual encounter in the last year?” “If yes, what type of protection?” “In every sexual encounter in the last year, did you always use a condom?” “If not, why not?”* Structured responses were provided for their reasons for inconsistent use of condoms. The first and second questions were used for validation purposes. Consistent condom use was derived using only the third questions.

#### Sexual behaviours

To measure participants’ sexual behaviours, we asked questions with dichotomised responses (yes/no) such as: “*Have you ever had sexual intercourse?” “If yes, with how many girls/boys have you ever had sex?”* Finally, we also asked questions about the most current or recent sexual behaviours of respondents by asking the following questions: *"In the past year, have you engaged in sex with anyone? If yes, with whom did you have sex (category of sexual partner, not name)? “In the last year, with how many girls/boys did you have sex?”* Three structured-responses were provided for participants to choose from, namely, “boyfriend/girlfriend only”, “casual partner only”, “both boyfriend/girlfriend and casual partners”.

#### Condom self-efficacy

Here, self-efficacy refers to the belief that one can successfully and confidently use condoms consistently and correctly [46]. Condom self-efficacy was measured with 13 questions adapted from a validated tool by Barkley et al. [46]. Self-efficacy was measured by asking respondents to indicate their levels of agreement on a 3-point Likert scale. Statements such as “I feel confident in my ability to put a condom on myself or my partner”, “I feel confident that I could purchase condoms without feeling embarrassed”, “I feel confident that I could remember to carry a condom with me, should I need one”, “I would not feel confident suggesting using condoms with a new partner because I would be afraid he or she would think I have a sexually transmitted disease” and “If I were to suggest using a condom to a partner, I would feel afraid that he or she would reject me”, among others, were used to elicit responses from participants. The responses were codified from 1 (‘Agree’) to 3 (‘Disagree’). The 13-item questions yielded a Cronbach’s alpha coefficient of 0.811, which demonstrated that they had a high degree of internal consistency. The 13 questions measuring self-efficacy were scored to yield a maximum score of 39, with higher scores indicating high condom self-efficacy and lower scores indicating lower condom self-efficacy.

#### Discussion of HIV/STIs with sexual partners

Discussion of HIV/STIs with sexual partners was assessed by asking the participants: have you discussed HIV/STIs with your sexual partner? Responses were a binary option of “Yes” or “No”.

#### Knowing a partner’s HIV status

Participants were asked to state whether they had knowledge of their partner’s HIV status. A binary response (Yes/No) was provided for participants to choose.

#### Covariates

Two main levels of covariate were included, which are demographic characteristics (such as age and sex) and substance use (use of substances such as alcohol, drugs and tobacco smoking). Participants reported their sex as either male or female. Participants also stated their age at their last birthday. Age was later categorised into less than 20 years, 20 to 24 years and above and 25 years and over. Alcohol use was assessed with three questions; have you ever drank alcohol? Do you currently drink alcohol? On how many days did you drink alcohol in the past month? Likewise, smoking behaviour was assessed by asking the participant three questions; have you ever smoked any tobacco product (cigarette, pipe, shisha)? Drug use was measured by asking participants to respond to three questions. First was "have you ever used substances/drugs like codeine, cannabis, tramadol etc. for pleasure or to ease tension? Also, we asked whether they currently used drugs like codeine, tramadol, cannabis etc. for pleasure or to ease tension. To assess the frequency of drug use among current users, we asked participants to state how many days in the last month did they use any of these drugs.

### Statistical analysis

The researchers used Statistical Package for Social Sciences (SPSS, version 24) to carry out descriptive statistical analyses on all variables. Specifically, mean was calculated for continuous variables and frequencies and percentages for categorical variables. We then used Chi-Square and Fisher exact test to examine the relationship between demographic variables, sexual behaviours, HIV testing, discussion of HIV/STDs, perceived risk of contracting HIV/STDs, condom self-efficacy and consistent condom use.

To test the study hypotheses, three models were fitted. The first, being the baseline model, was used to estimate the independent effect of each main predictor variable on consistent condom use. Model 2 controlled for demographic covariates such as age and sex, while Model 3 included both demographic and substance use covariates as controls. Due to the many hypotheses of the study, adjusted and unadjusted binary logistic regression models were used to determine the main predictors of consistent condom use. There was collinearity between ‘currently smoke’ and ‘drug use’ thus; currently smoke was not included in the adjusted model. The analysis was done at a 95% confidence level, and an alpha value of less than 0.05 was deemed statistically significant. List-wise deletion of cases with missing responses (those not sexually active in the previous year) led to only 498 participants being involved in the logistic regression.

## Results

### Descriptive results

As stated above, the analysis was based on the 498 participants who reported being sexually active in the past year. The median age of study participants was 22 years. Most of the participants were below 25 years of age (71.9%), resided in off-campus residences (82.3%) and shared their rooms with at least one person (66.5%). Just over half of them had ever taken an HIV test (Table 1). The minimum and maximum condom self-efficacy scores were 13 and 39, respectively. The median score was 29. The mean score was 28.96 (SD ± 5.97). The mean score for males and females were 30.31 (SD ±5.69) and 27.48 (SD ±6.22), respectively. Male students had a significantly higher condom self-efficacy score compared to female students (Mean difference = 2.58; CI = 1.53–3.64). For further analysis, we dichotomised the condom self-efficacy scores into low and high scores, with a score of 27–39 considered high and a score below 27 considered low. The cutoff score of 27, considered to be high condom self-efficacy score, is based on the fact that such participant must have responded affirmatively to 9 out of the 13 questions. In other words, such participants have a score of 70%. Over 66% of the participants were in the high condom self-efficacy category, with a significant sex variation (male 74.6% vs female 56.5%). Less than half of the students (42.8%) knew their partner’s HIV status. There was no significant difference between sex and knowing one’s partner HIV status (Additional file 1: Table S1). Only 42% of the students had discussed HIV/STIs with their sexual partners, with no significant sex variation.

**Table 1:** Demographic characteristics of sexually active participants

Variables	Frequency (*N* = 498)	Percentage
Sex		
Male	263	52.8
Female	235	47.2
Age Category		
Less than 20	94	18.9
20–24	264	53.0
Above 24	140	28.1
Years in the university		
First	113	22.7
Second	123	24.7
Third	107	21.5
Fourth	115	23.1
Fifth	28	5.6
Sixth	12	2.4
Residence		
Campus residence	88	17.7
Off-campus residence	410	82.3
Current alcohol users	188	37.8
Current smokers	106	21.3
Current drug users	120	24.1

Over half (56.1%) of the 498 had had sex with only their girl/boyfriends, 20.5% with casual partners only, 3.7% with commercial sex workers only and 19.7% with both girl/boyfriends, casual partners and commercial sex workers. The median number of sexual partners was 2. The minimum and the maximum number of sexual partners in the previous year were 1 and 15, respectively.

As shown in Additional file 1: Table S1, only 38.6% (95% CI: 34.3–43.0%) of sexually active students used condoms consistently in the previous year. A higher proportion of males (41.8% CI 35.8–48.0%) compared to females (34.9% CI 28.8–41.4%) affirmed that they consistently used condoms in the past year.

### Multivariable analysis

The results of our hypotheses on the relationship between condom self-efficacy, knowing one’s partner HIV status, partner communication about HIV/STIs, number of sexual partners and location of residence and consistent condom use are presented in Table 2.

**Table 2:** Binary logistic regression models showing relationship between self-efficacy, discussion of HIV/STIs with partner, knowing partners HIV status, schooling in high HIV prevalence area, types of sexual partners and consistent condom use

Variables	Model 1	Model 2	Model 3
	UOR (95%CI)	UOR (95%CI)	UOR (95%CI)
Condom self-efficacy			
High self-efficacy score ≥ 27	2.45 (1.63–3.68)***	2.40 (1.59–3.64)*	2.40 (1.58–3.64)***
Low self-efficacy score < 27	1	1	
Discussed HIV/STDs with sexual partner			
Yes	1.90 (1.31–2.74)*	2.03 (1.38–2.98)***	1.91 (1.29–2.83)*
No	1	1	1
Know sexual partner HIV status			
Yes	1.51 (1.05–2.17)*	1.56 (1.08–2.26)*	1.48 (1.02–2.16)*
No	1	1	1
University location			
Located in high HIV prevalence area	2.84 (1.95–4.13)***	3.04 (2.07–4.48)***	2.86 (1.92–4.28)***
Located in low HIV prevalence area	1	1	1
Type of with sexual partner/s in last year preceding the survey			
Main partner only (Boyfriend/girlfriend)	1.68 (1.16–2.43)*	1.82 (1.24–2.67)*	1.74 (1.17–2.60)*
Boyfriend/girlfriend, casual partner and commercial sex workers	1	1	1

Model 1- contains no covariates

Model 2- controlled for demographic characteristics

Model – controlled for demographic characteristics and established risk factors for risky sexual behaviour such as alcohol and drug use

*P-value < 0.05, *** *P*-value < 0.001, *UOR* unadjusted odds ratio, *AOR* adjusted odds ratio, *CI* confidence interval

To test hypothesis 1, which examined the relationship between condom self-efficacy and condom use, we fitted three models. The first is the baseline model that estimated the independent net effect of condom self-efficacy on consistent condom use. The second model controlled for demographic characteristics such as age and sex. The third model added both demographic and substance use covariates. The results indicate that condom self-efficacy is positively associated with consistent condom use. The direction and magnitude of effect remain even after adding the demographic and substance use covariates, as shown in model three. Overall, the results indicate that high condom self-efficacy is associated with a higher likelihood of consistent condom use.

On the relationship between consistent condom use and knowing one’s partner status, our hypothesis two, we fitted three models and control for demographic and behavioural covariates. The analysis reveals that knowing one’s sexual partner’s HIV status increases the odds of consistent condom use. The direction and magnitude of effect persist after controlling for demographic and behavioural covariates. Our finding on the third hypothesis shows that discussion of HIV/STIs with a sexual partner is associated with a higher likelihood of consistent condom use. The magnitude and direction of effect remain after controlling for demographic and substance use covariates.

We likewise fitted three models to understand the relationship between residing in a high HIV prevalence area compared to residing in a low HIV prevalence area. The results show that students studying in a high HIV prevalence area were about three times more likely to consistently use condoms compared to those in a low HIV prevalent area. Overall the finding is robust as evident in model three results.

Our last hypothesis focused on the association between relationship types and consistent condom use. We found that students in a steady relationship or who had sex with only their boyfriend/girlfriend had a higher odds of consistent condom use compared with those who had sex with both their girlfriend/boyfriend and casual partners.

We also examined the main predictors of consistent condom use by including all the main independent variables, and the demographic and substance use covariates in the adjusted binary logistic regression model (Table 3). After adjusting for confounding variable (smoking), only condom self-efficacy, schooling in a high HIV prevalence area, male sex, and having only one sexual partner in the last 1 year were significantly associated with consistent condom use.

**Table 3:** Adjusted and unadjusted logistic regression analysis showing predictors of consistent condom use

Variables	UOR (95%CI)	AOR (95%CI)
Self-efficacy		
High self-efficacy score ≥ 27	2.45 (1.63–3.68)***	1.86 (1.19–2.91)*
Low self-efficacy score < 27	1	1
University location		
Located in a high HIV prevalence area	2.84 (1.95–4.13)***	2.11 (1.34–3.32)*
Located in a low HIV prevalence area	1	1
Discussed HIV/STDs with sexual partner		
Yes	1.90 (1.31–2.74)*	1.37 (0.85–2.24)
No	1	1
Know partner’s HIV status		
Yes	1.51 (1.05–2.17)*	1.04 (0.65–1.65)
No	1	1
Type of with sexual partner/s in last year preceding the survey		
Boyfriend/girlfriend only	1.68 (1.16–2.43)*	0.86 (0.51–1.42)
Boyfriend/girlfriend, casual partner and commercial sex workers	1	1
Number of sexual partners in the last year		
Only one sexual partner	2.08 (1.44–3.0)***	1.99 (1.24–3.19)*
More one sexual partners	1	1
Age category		
Less than 20 years	0.92 (0.54–1.57)	1.54 (0.83–2.85)
20–24 years	0.97 (0.64–1.48)	1.09 (0.70–1.71)
Above 24 years	1	1
Sex		
Male	1.34 (0.93–1.93)	1.53 (1.01–2.32)*
Female	1	1
Current alcohol users		
Yes	0.95 (0.65–1.38)	1.30 (0.81–2.10)
No	1	1
Currently use drug		
Yes	0.58 (0.37–0.90)*	0.77 (0.44–1.33)
No	1	1

*P-value < 0.05, *** *P*-value < 0.001

#### Reasons for inconsistent condom use

Finally, we probed the reasons for inconsistent condom use, and as shown in Table 4, trust, unavailability of condoms, dislike of condoms and a perception that condoms reduce sexual pleasure were the main reasons for inconsistent use of condoms among the study participants.

**Table 4:** Reasons for inconsistent condom use

Reasons	Frequency (*n* = 291)	Percent
I trust my partner	79	27.1
Condom reduces fun	75	25.8
I don’t like condoms	64	22.0
I cannot afford it	3	1.0
There was no condom available when I need to use it	70	24.1

## Discussion

This study determined the rate of consistent condom use, explored the determinants of condom use consistency and explored reasons for inconsistent condom use in a sample of young men and women enrolled at two Nigerian universities. Our analysis revealed that only 38.6% of the students consistently used condoms in the previous year. The level of condom use consistency reported in our study is higher than those reported in previous studies with similar demographics in Nigeria. For instance, a lower rate of condom use consistency (24.2%) was reported in a study conducted in a tertiary institution in Ile-Ife, Southwestern, Nigeria [39]. An even lower rate of consistent condom use (15% for men and 4% for women) was reported among fresh graduates of tertiary institutions who are on migration for compulsory national assignment in Ibadan, Nigeria [30]. However, a higher rate of condom use consistency (48.8%) was reported among HIV positive individuals attending an HIV treatment centre in Lagos, Nigeria [38]. The rate of consistent condom use reported in this study is within the range reported among undergraduate students in previous studies (29.6–52.4%) [9, 33, 47].

The findings of the present study revealed that the level of consistent condom use is low among Nigerian university students. Considering that many of these students had multiple sexual partners in the year preceding the study, inconsistent condom use among Nigerian students is worrisome and clearly indicates a need for a public health intervention. The findings thus have broad implications for the sexual health of Nigerian university students. Inconsistent use of condoms, especially with multiple sexual partners, is a risky sexual behaviour with dire consequences. Beyond the risk of HIV and other STI transmission, students could also be faced with associated unplanned pregnancies and unsafe abortions [17, 48, 49]. Counselling students on consistent condom use, including assessment of self-efficacy towards condom use, would be an important step towards addressing these public health issues in the study setting. This finding further lays credence to studies that have shown that unprotected sex is common among Nigerian students [3, 50, 51].

Consistent with previous studies [10, 13, 41] our results strongly support the hypothesis that condom self-efficacy is associated with a higher likelihood of consistent condom use. Despite controlling for relevant covariates, the magnitude and direction of effect of condom self-efficacy on consistent condom use persist. Individuals with high condom self-efficacy scores were approximately twice as likely to report consistent condom use compared to those with low condom self-efficacy scores. Although our study is cross-sectional, nonetheless the finding suggests that intervention that targets increasing condom self-efficacy among students could be useful in increasing the rate of consistent condom use in the study setting. Social marketing campaigns on campuses and motivational counselling at campus-based clinics are cheap interventions that could be implemented on campuses across the country.

One interesting finding of this study is that discussion of HIV/STDs with sexual partners and knowing one’s partner HIV status were associated with consistent condom use. This suggests that encouraging youths to have HIV/STD conversations with sexual partners could be a useful intervention for increasing the level of consistent condom use. In other words, the discussion on the risk of contracting HIV/STDs could promote the consistent use of condoms. A study conducted in South Africa found that individuals who discussed HIV/STDs with their sexual partners were more likely to use condoms compared to respondents who had not [52].

This study also established that students studying in a high HIV prevalence area had higher odds of consistent condom use. This finding is expected, considering that interventions aimed at reducing HIV transmission are concentrated in these areas. It is plausible that people are more aware of their risk of contracting HIV and ways of preventing HIV transmission. Also, in areas with a concentration of HIV risk-reduction interventions, condoms are more likely to be supplied freely. Considering that this study also found that lack of condom availability is among the reasons for inconsistent condom use, one could, therefore, infer that people are more likely to use condoms in areas targeted with risk reduction interventions because of condom availability. This finding may also be construed as evidence of the effectiveness of the HIV risk reduction interventions targeting areas with high HIV prevalence in Nigeria.

This study also shows that having just one sexual partner or only the partner in the past year is associated with a higher likelihood of consistent condom use. This finding corroborates a study [41] that found an association between consistent condom use and having just one partner among rural young women in South Africa. However, one Nigerian study reported that women with multiple partners were more likely to consistently use condoms [39] while having multiple sexual partners did not significantly affect consistent condom use among men [40]. In one Kenyan study, men mostly used condoms when having sex with commercial sex workers and were less likely to use condoms when having sexual intercourse with regular partners and casual partners [53]. It could be that individuals who maintain a steady relationship are more likely to engage in protective sexual behaviour, including consistent condom use. In other words, those that engage in sex with both casual partners and main partners are more likely to engage in risky sexual behaviour, including inconsistent condom use compared to those that did not. It is also possible that those who engaged in sex with both casual partners and steady partner would use condoms with casual partners and fail to use condoms with a steady partner.

Trust, unavailability of condoms, dislike of condoms and perceptions that condoms reduce sexual pleasure were the main reasons for inconsistent use of condoms among students – all of which corroborated previous studies [11–13, 29, 30]. Also, a previous study conducted among Nigerian university students has shown that despite being aware of the risks associated with unprotected sex, Nigerian students engage in risky sexual behaviour due to perceptions that condoms reduce sexual pleasure and the often unfounded belief that their partners are free of any sexually transmitted diseases [3]. This study contributes to the extant literature by adding that, unavailability of condoms at the time they are needed, as well as a personal dislike of condoms, are among the reasons for inconsistent condom use among Nigerian university students. Provisioning of condoms on campuses would be an important intervention to address the unavailability of condoms and could increase the level of consistent condom use among university students. Given that this intervention is cheap and has been effectively implemented in other settings such as South African campuses, government and non-governmental agencies should prioritise this intervention. Also, it is worth noting that the use of oral pills, implants, injectables and IUD is very low among young people in Nigeria, and this is as a result of several misconceptions regarding these contraceptive methods [54]. In this study, for example, no student reported the use of these contraceptive methods. Young people in Nigeria believe women that have no children should not use hormonal contraceptive methods and often considered there use to lead to future infertility [50, 51].

### Limitations

Although this study elevates the discourse on consistent condom use, the findings must be generalised with caution. This study was conducted among young adults with higher education levels compared to the general population, thus limiting the generalisability of its findings to the overall Nigerian population. Also, the use of a cross-sectional design in this study indicates that the association between condom self-efficacy and consistent condom use does not infer causation. Also, due to the reliance on self-reporting of consistent condom use, one cannot exclude the possibility of under-reporting of inconsistent condom use as a result of social desirability bias. It is also important to note that people with multiple partners may not use condoms with their regular partner but use condoms with their casual partners.

## Conclusion

This study shows that the level of consistent condom use is low among university students in Nigeria. High condom self-efficacy, type of sexual partner, and having discussed HIV/STDs with sexual partners were significantly associated with consistent condom use. Counselling young adults in Nigeria on condom self-efficacy, and encouraging the discussion of sexually transmitted infections with sexual partners are central to improving the level of consistent condom use among Nigerian university students. Intervention such as the provisioning of condoms to students on campus could help in addressing the unavailability barrier to consistent condom use and increase the level of consistent condom use among Nigerian university students. Besides intervention to improve condom self-efficacy, regular HIV testing and awareness and use of pre-exposure prophylaxis are among effective intervention for prevention of HIV and STIs, especially for individuals who find condoms to be inappropriate in their relationship.

## Additional file

Additional file 1: **Table S1.** Demographic and behavioural correlates of consistent condom use. CI-confidence interval (DOCX 17 kb)

## Data Availability

All relevant data generated during this study will be made available by the corresponding author upon reasonable request.

## Abbreviations

HIV: Human immunodeficiency virus
STDs: Sexually transmitted diseases
STIs: Sexually transmitted infections
