# Supplementary material for: Factors associated with consistent condom use: a cross-sectional survey of two Nigerian universities
Source: BMC Public Health. 2019 Sep 2;19:1207. doi: 10.1186/s12889-019-7543-1 (PMC6719351; doi:10.1186/s12889-019-7543-1)
Supplement: Supplementary file 1 — Table S1. Demographic and behavioural correlates of consistent condom use. CI-confidence interval (DOCX 17 kb) [file 12889_2019_7543_MOESM1_ESM.docx]

| **Variables** | **Consistent condom users**  **N (%)** | **95% CI** | **Inconsistent condom users**  **N (%)** | **95% CI** | ***P*** |
| --- | --- | --- | --- | --- | --- |
| Overall | 192 (38.6) | 34.3-43.0 | 306 (61.4) | 57.0-65.7 |  |
| Age category |  |  |  |  |  |
| Less than 20 years | 35 (37.2) | 27.5-47.8 | 59 (62.8) | 52.2-72.5 | 0.951 |
| 20-24 years | 102 (38.6) | 32.7-44.8 | 162 (61.4) | 55.2-67.3 |  |
| 25 years and over | 55 (39.3) | 31.1-47.9 | 85 (60.7) | 52.1-68.9 |  |
| Sex |  |  |  |  |  |
| Male | 110 (41.8) | 35.8-48.0 | 153 (58.2) | 52.0-64.2 | 0.113 |
| Female | 82 (34.9) | 28.8-41.4 | 153 (65.1) | 58.6-71.2 |  |
| Current alcohol users |  |  |  |  |  |
| Yes | 71 (37.8) | 30.8-45.1 | 117 (62.2) | 54.9-69.2 | 0.427 |
| No | 121 (39.0) | 33.6-44.7 | 189 (61.0) | 55.3-66.4 |  |
| Current smokers |  |  |  |  |  |
| Yes | 31 (29.2) | 20.8-38.9 | 75 (70.8) | 61.1-79.2 | 0.017 |
| No | 159 (41.0) | 36.0-46.1 | 229 (59.0) | 53.9-64.0 |  |
| Current drug users |  |  |  |  |  |
| Yes | 35 (29.2) | 21.2-38.2 | 85 (70.8) | 61.8-78.8 | 0.010 |
| No | 157 (41.5) | 36.5-46.7 | 221 (58.5) | 53.3-63.5 |  |
| Discussed HIV/STDs with sexual partner |  |  |  |  |  |
| Yes | 99 (47.4) | 40.4-54.4 | 110 (52.6) | 45.6-59.6 | <0.001 |
| No | 93 (32.2) | 26.8-37.9 | 196 (67.8) | 62.1-73.2 |  |
| Know partner’s HIV status |  |  |  |  |  |
| Yes | 94 (44.1) | 37.4-51.1 | 119 (55.9) | 48.9-62.6 | 0.017 |
| No | 98 (34.4) | 28.9-40.2 | 187 (65.6) | 59.8-71.1 |  |
| University location |  |  |  |  |  |
| Located in high HIV prevalence area | 126 (50.6) | 44.2-57.0 | 123 (49.4) | 43.0-55.8 | <0.001 |
| Located in low HIV prevalence area | 66 (26.5) | 21.1-32.4 | 183 (73.5) | 67.6-78.9 |  |
| Type of with sexual partner/s in last year preceding the survey |  |  |  |  |  |
| Boyfriend/girlfriend only | 122 (43.9) | 38.0-49.9 | 156 (56.1) | 50.1-62.0 | 0.004 |
| Boyfriend/girlfriend, casual partner and commercial sex workers | 70 (31.8) | 25.7-38.4 | 150 (68.2) | 61.6-74.3 |  |
| Number of sexual partners in the last one year |  |  |  |  |  |
| One only | 115 (47.3) | 40.9-53.8 | 128 (52.7) | 46.2-59.1 | <0.001 |
| Two and more | 77 (30.2) | 24.6-36.2 | 178 (69.8) | 63.8-75.4 |  |
| Self-efficacy score |  |  |  |  |  |
| Low self-efficacy score <27 (ref) | 44 (25.4) | 19.1-32.6 | 129 (74.9) | 67.4-80.9 | <0.001 |
| High self-efficacy score ≥27 | 148 (45.5) | 40.0-51.1 | 177 (54.5) | 48.9-60.0 |  |
